# Serum amyloid A and fibrinogen as markers for early detection of surgical site infection associated with internal fixation in the horse

**DOI:** 10.3389/fvets.2022.960865

**Published:** 2022-10-10

**Authors:** Catherine C. Thurston, Darko Stefanovski, Melissa C. MacKinnon, Hannah-Sophie Chapman, Dean W. Richardson, David G. Levine

**Affiliations:** ^1^New Bolton Center, Department of Clinical Studies, University of Pennsylvania, Kennett Square, PA, United States; ^2^Department of Population Medicine, University of Waterloo, Waterloo, ON, Canada; ^3^Pferdeklinik Leichlingen, Leichlingen, Germany

**Keywords:** serum amyloid A, postoperative infection, fibrinogen, surgical site infection, equine surgery, internal fixation, equine orthopedics, fracture repair

## Abstract

The objective of this study was to determine the diagnostic ability of serum amyloid A (SAA) and fibrinogen for early detection of surgical site infection (SSI) after equine internal fixation. Horses undergoing internal fixation for fracture, arthrodesis, or osteotomy with internal fixation for limb deformity were included in the study. SAA and fibrinogen were measured on blood samples preoperatively and on days 1, 3, 5, 7, 10, and 14 postoperatively. Statistical analysis included use of Spearman's rank correlation, logistic regression, and calculating the area under the receiver operating characteristic (ROC) curve. SAA and fibrinogen measurements were both associated with SSI, with SAA being considered an excellent marker (area under the ROC curve 0.8) and fibrinogen being considered acceptable (<0.8). As the amount of time postoperatively increased, SAA elevations indicated a higher likelihood of SSI (area under the ROC curve 0.8 compared with fibrinogen 0.7). SAA and fibrinogen were predictive markers of SSI and SAA is of greater diagnostic utility when compared with fibrinogen. Persistent elevations of SAA postoperatively are associated with the development of SSI. Serial monitoring of SAA can be used to help predict the development of SSI in horses undergoing internal fixation. This may lead to earlier suspicion, and therefore recognition and treatment of SSI.

## Introduction

Surgical site infection (SSI), in particular implant infection, is the most significant complication in equine long bone fracture repair and is associated with an increase in morbidity and mortality. Rates of SSI have been reported to be 14 to 28%, and horses that develop SSI are 12.34 times more likely not to survive to hospital discharge (1, 2). Prompt diagnosis is critical as early aggressive therapy is thought to be necessary for treatment. Serum amyloid A (SAA) is an apolipoprotein produced by the liver in response to inflammation and is a major acute phase protein in the horse (3, 4). It is present in low levels in a disease-free state, increases by up to 1,000-fold during the acute phase response, and returns to normal levels with resolution of inflammation or infection (3, 4). Fibrinogen in contrast is a minor acute phase protein that is always present in blood samples, increases by 1-2 times with an inflammatory stimulus, and whose changes lag behind resolution of inflammation or infection (3). SAA has shown promise as a sensitive marker of infection in the horse in several different disease states including synovial infection, viral and bacterial respiratory infection, and gastrointestinal disorders as well as in experimental models of disease states in the horse (5–22).

The response of SAA following different surgical procedures such as arthroscopy, upper airway surgery, and abdominal surgery has been documented and indicates an expected peak postoperatively that varies in its value based on degree of surgical trauma (23–27). To our knowledge, an association between SAA and SSI after internal fixation in horses has not been reported. Fibrinogen is currently the most frequently used marker for postoperative orthopedic infection, but changes lag behind clinical signs of disease in the authors' experience. Changes in SAA levels occur faster than changes in fibrinogen levels indicating that it may be a better clinicopathologic parameter to monitor in the postoperative period to aid in early diagnosis of SSI.

The objective of this study was to evaluate the diagnostic ability of SAA and fibrinogen in predicting the occurrence of SSI in equine fracture repair, arthrodesis, and internal fixation for limb deformity. We hypothesize that SAA and fibrinogen will be predictors of the early development of SSI in horses undergoing internal fixation for long bone fracture repair, arthrodesis, or osteotomy for limb deformity. In addition, we hypothesize that SAA will be of a greater diagnostic utility for SSI than fibrinogen.

## Materials and methods

This study received owner consent and approval from the Institutional Animal Care and Use Committee of the University of Pennsylvania New Bolton Center. Horses from 2014 to 2021 undergoing internal fixation were enrolled prospectively in the cohort study. This included horses with long bone fracture, horses requiring arthrodesis due to osteoarthritis or fracture, and horses with limb deformities requiring osteotomy and internal fixation. Horses with simple fractures repaired with cortical screws placed in lag fashion were excluded. Signalment (age, sex, breed), anatomic location of the lesion, fracture configuration, and development of SSI were recorded. Diagnosis of SSI was made by the one of four (4) surgeons, who were blinded to SAA values, based on one or more of the following criteria: the presence of persistent drainage/draining tracts, increased degree of lameness, increased rectal temperature, radiographic or ultrasonographic evidence of infection, or positive bacterial culture from the surgical site. Surgeons followed up on cases by communication with the referring veterinarian and/or owner for at least 8 weeks. Any horse with signs of infection was readmitted to the hospital; therefore, final diagnosis of SSI was always made by the surgeon(s).

SAA and plasma fibrinogen levels were measured preoperatively (day 0) and on days 1, 3, 5, 7, 10, and 14 postoperatively. SAA was measured on whole blood using the Stablelab^®^ EQ-1 Handheld Reader (Zoetis, Parsippany, New Jersey). Fibrinogen was measured quantitatively on citrate plasma using the ACL Elite Coagulation Analyzer (Werfen, Bedford, ME). Samples were stored at 37°F and were analyzed the same day as collection. Elevations in SAA were defined as >20 ug/mL (11). The reference range for normal fibrinogen was 200–400 mg/mL.

All analyses were conducted with Stata 16MP, StataCorp, State College TX, with two-sided tests of hypotheses and a *p*-value < 0.05 as the criterion for statistical significance. Descriptive analyses included the reporting of frequency counts and percentages for categorical variables. Continuous variables were reported as medians (with their respective range). Normality of the data was assessed using Shapiro-Wilk test.

Inference statistical analysis was conducted in two steps. First, an exploratory univariate analysis using Spearman rank correlation was conducted to identify whether fibrinogen and SAA were showing statistical trends (*P* < 0.2) of association with outcome of interest (SSI). Second and final, the independent variables identified to show trend of association with SSI were further quantified using logistic regression where the time (in days) when the samples were acquired was used as confounder. There were two separate sets of logistic regression models considered. The first logistic models were simple had only one fixed effect (SAA or fibrinogen) as continuous independent variable. The second set included time as continuous variable as a confounder. Subsequently, test of equality of receiver operating characteristic (ROC) areas was used to assess whether one of the independent variables (fibrinogen or SAA) was significantly better than the other. Sensitivity and specificity of a diagnostic procedure rely on a single cut point to assess the classification accuracy of the diagnostic test. The area under the curve of the receiver operating characteristics (AUC ROC) is a more complete description of the classification accuracy. The AUC ROC varies from 0 to 1 with values closer to 1 indicating an outstanding classification accuracy. Thus, it provides a measure of the logistic model's ability to discriminate between animals with and without SSI (28). The ROC figure shows the curve where for each value of the cutoff ranging from 0 to 1, the value of the sensitivity vs. the 1-specificity is plotted. The test of equality of the ROC test two factors (in our case this is fibrinogen and SAA) against the reference variable to show if these ROC are equal or different (29). Cutoff values for both SAA and fibrinogen were calculated and both sensitivity and specificity for those cutoff values were calculated.

## Results

Thirty-nine horses were included over a 7-year period. One horse was included in the study twice, 4 years apart, for two separate surgical procedures, for a total of 40 procedures. Horses were a median age of 6 years old (range of 1 week to 20 years). Eighteen mares, 16 geldings, and 5 intact males were included. Breeds included Thoroughbreds (17), Warmbloods (11), Standardbreds (2), Miniature Horses (2), Hackney Ponies (2), Quarter Horse (1), Arabian (1), Paint (1), and Arabian cross (1). Breed was not available for one horse. Surgical procedures performed included proximal interphalangeal arthrodesis with plate(s) and screws (12), metacarpophalangeal/metatarsophalangeal arthrodesis (10), internal fixation of third metacarpal/metatarsal bone fracture (5), proximal phalangeal fracture (3), tibial fracture (3), femoral fracture (1), scapular fracture (1), olecranon fracture (1), distal interphalangeal arthrodesis (1), tarsometatarsal arthrodesis (1), pancarpal arthrodesis (1), and radial osteotomy and internal fixation for rotational limb deformity (1). The median and range values for SAA are summarized in Table 1. The median and range values for fibrinogen are summarized in Table 2. SSI developed in 12/40 procedures (30%). Discharge from the surgical site occurred in 12 cases (100%), increased degree of lameness occurred in 11 cases (92%), positive culture was obtained in 6 cases (50%), fever developed in 4 cases (33%), radiographic evidence of SSI was present in 3 cases (25%), and ultrasonographic evidence of SSI was present in 0 cases (0%). Median number of days to diagnosis of SSI was 7.5 days (range 1-39). The median value of SAA peaked on day 3 and decreased on day 5 and again on day 7 in cases without infection whereas the median SAA value continued to rise or plateaued in infected cases after day 3.

**Table 1:** Median and range serum amyloid A values for each postoperative day.

**Day**	**0**	**1**	**3**	**5**	**7**	**10**	**14**
No SSI	0 (0-910) [27]	159 (0-1897) [25]	346 (0-2635) [24]	275 (0-1663) [20]	56 (0-723) [10]	55.5 (0-117) [5]	2 (1-3) [2]
SSI	1 (0-1234) [11]	358 (7-1124) [10]	955 (1-2389) [11]	1011 (3-1887) [12]	1207 (2-2996) [5]	1170.5 (378-1844) [4]	894.5 (134-1655) [2]

Range is listed in parentheses () and number of samples listed in brackets [ ]. Surgical site infection (SSI).

**Table 2:** Median and range fibrinogen values for each postoperative day.

**Day**	**0**	**1**	**3**	**5**	**7**	**10**	**14**
No SSI	343(235-1,062) [28]	427 (182-1,064) [25]	508 (295-1,108) [26]	544 (372-890) [21]	623 (404-800) [10]	569 (497-697) [4]	716 (702-730) [2]
SSI	336 (238-894) [11]	481 (397-640) [11]	517 (364-857) [11]	624.5 (347-1,178) [12]	848 (502-1,166) [6]	878 (504-1,126) [4]	1,083.5(474-1,420) [2]

Range is listed in parentheses () and number of samples listed in brackets []. Surgical site infection (SSI).

Spearman's rank correlation coefficient (rho) was 0.47 for SAA, and 0.32 for fibrinogen with *P*-values of < 0.001 for both values, indicating that both SAA and fibrinogen are associated with SSI. Logistic regression resulted in a Pseudo r2 value of 0.18 with *P* < 0.001 indicating a significant association between SAA value and SSI.

**Figure 1:**
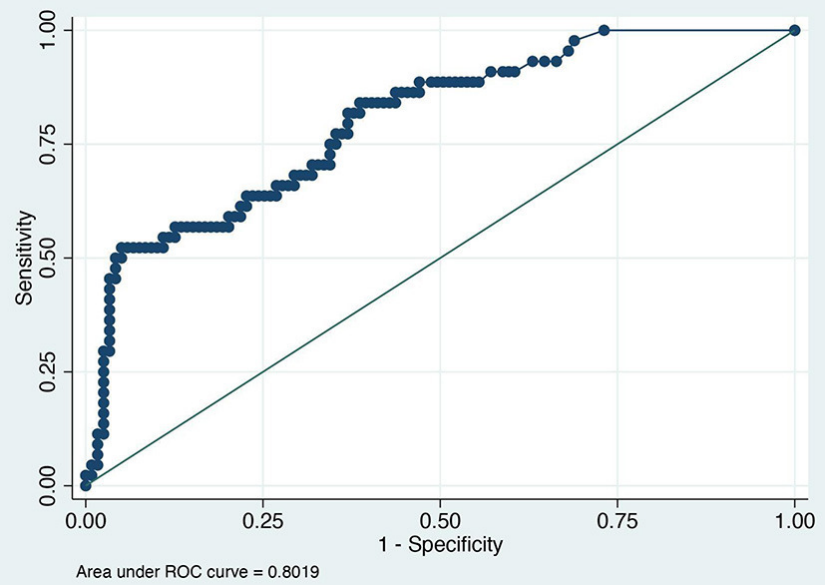
Receiver operator characteristic (ROC) curve for SAA. Each blue dot indicates different cutoff probability (varying from 0 to 1) value for which the sensitivity on the y-axis and the (1-specificity) on the x-axis was estimated using a multivariable logistic model including SAA and day when the sample was acquired as independent variables.

**Figure 2:**
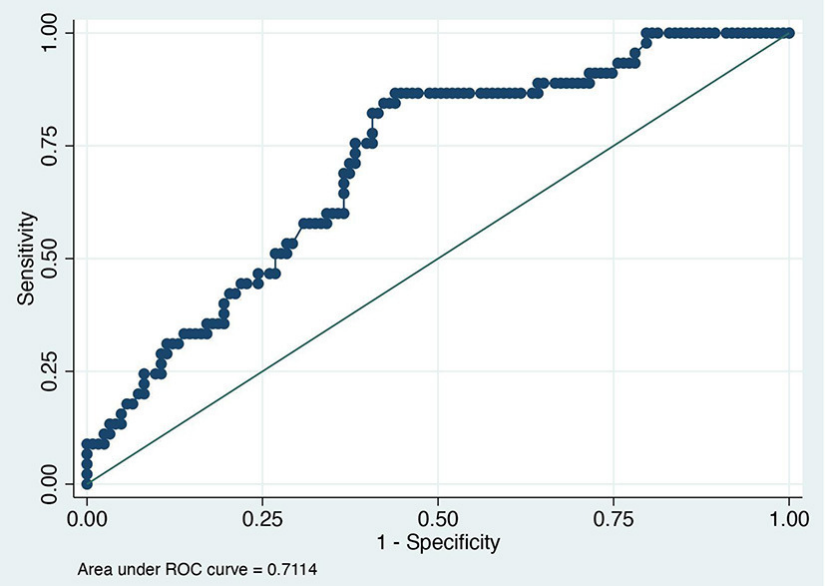
Receiver operator characteristic (ROC) curve for fibrinogen. Each blue dot indicates different cutoff probability (varying from 0 to 1) value for which the sensitivity on the y-axis and the (1-specificity) on the x-axis was estimated using a multivariable logistic model including fibrinogen and day when the sample was acquired as independent variables.

**Figure 3:**
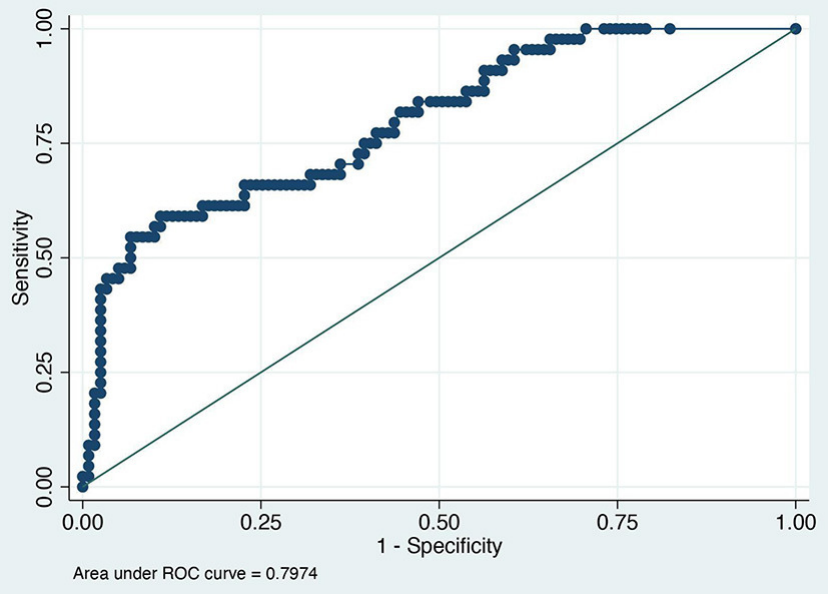
Receiver operator characteristic curve for surgical site infection and serum amyloid A normalized for postoperative day.

**Figure 4:**
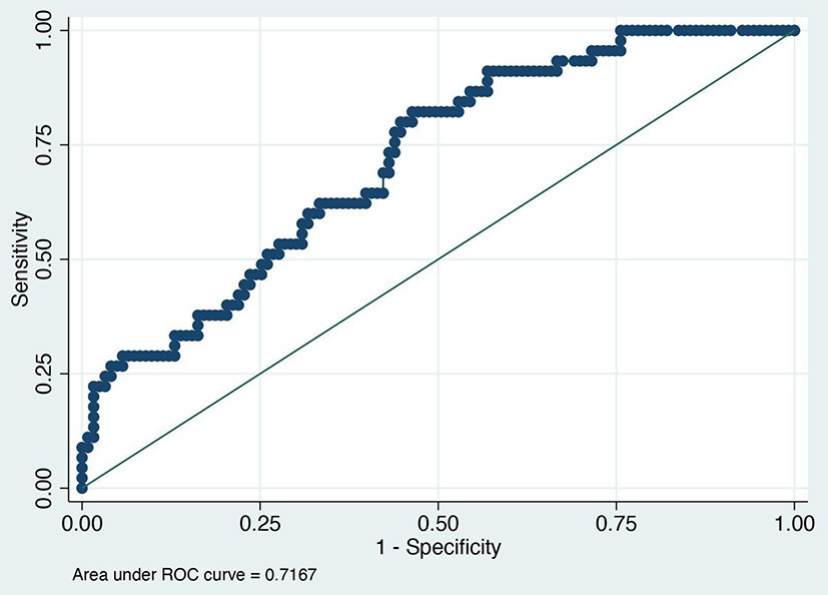
Receiver operator characteristic curve for surgical site infection and fibrinogen normalized for postoperative day.

The value of the AUC ROC curve for the gold standard for SAA as the only independent variable was 0.8 (95% CI: 0.7–0.9) indicating that it is an excellent marker for SSI (Figure 1) and for fibrinogen as the single predictor was 0.7 (95% CI: 0.6–0.8) which is considered acceptable (Figure 2); this shows that SAA was a better indicator of SSI with significantly higher (P=0.03) AUC ROC. Based on a univariate linear regression, it was established that the concentration increases in linear fashion with increase of time. Hence, the time (as a continuous variable) was included as a significant confounder in both models that predicted the likelihood of SSI. When the AUC ROC was calculated for SAA with day of sample collection as a confounder, the value was 0.8 (95% CI: 0.7–0.9) (Figure 3), indicating that as the amount of time postoperatively increased, SAA elevations indicated a higher likelihood of SSI. The same analysis for fibrinogen produced an AUC ROC of 0.7 (95% CI: 0.6–0.8) (Figure 4) indicating that it does predict SSI, but not as well as SAA. Ten of 12 horses (83%) that developed SSI had persistently elevated SAA levels on day 5 and beyond.

The cutoff value established for SAA was 1,000 ug/mL at any time point, meaning that values above 1,000 indicated the presence of SSI with a sensitivity of 67% and specificity of 56% (Table 3). The cutoff value established for fibrinogen was 700 mg/mL at any time point, meaning that values above 700 indicated the presence of SSI with a sensitivity of 58% and specificity of 51% (Table 4). For the multivariable models we were not able to produce a cutoff value because the likelihood of SSI is also dependent on the time the sample was obtained besides the values of SAA and fibrinogen.

**Table 3:** Serum amyloid A (SAA) values of samples corresponding to the presence or not of surgical site infection (SSI).

**SAA value**	**No SSI**	**SSI**	**Total**
<1,000 ug/mL	113	21	134
>1,000 ug/mL	89	51	140
Total	202	72	274

**Table 4:** Fibrinogen values of samples corresponding to the presence or not of surgical site infection (SSI).

**Fibrinogen value**	**No SSI**	**SSI**	**Total**
<700 mg/mL	103	30	133
>700 mg/mL	99	42	141
Total	202	72	274

## Discussion

We confirmed our hypotheses that SAA and fibrinogen are early predictors of the development of SSI in horses undergoing long bone fracture repair or arthrodesis and that SAA has a greater diagnostic utility than fibrinogen. SAA has been shown to have a predictable response to surgical trauma in elective surgical cases with peak levels being reached between 36–72 h postoperatively and returning to baseline by 7–8 days postoperatively in cases where no postoperative complications were seen (23, 25). In a study looking at horses undergoing routine castration, all horses in both the group with complications and the group without had an increased SAA on day 3 postoperatively (25). The horses without SSI had an SAA commensurate with preoperative levels on day 8 postoperatively and the group with SSI had increased SAA levels at day 8. We found similar results in our case population. In most cases without infection in the current study, as evidenced by the provided median values, SAA peaked on day 3 postoperatively, and began decreasing on day 5 or 7. This peak followed by decline was not seen in cases that went on to develop SSI. The monitoring of the trend of SAA values has been previously described in horses with experimentally induced septic synovitis (21). In this model, synovial sepsis was determined to be resolved at day 4, plasma SAA began to decline daily after a peak on day 3, but remained elevated until day 9 or 10, also similar to our cases (21). SAA has been shown to increase commensurately with the degree of surgical trauma (26). Therefore, cases that underwent open reduction and internal fixation may have had higher SAA values than those undergoing closed reduction and internal fixation with minimally invasive techniques used; this was not analyzed in this study (26). Higher SAA and fibrinogen values may have been a result of the degree of surgical trauma induced by a particular repair rather than the presence of infection which may have confounded the results of this study. Conflicting reports exist in the literature regarding the effects of general anesthesia on serum SAA levels; therefore, general anesthesia's effect on the results of this study are unknown (30, 31).

Although this report establishes cutoff values for SAA and fibrinogen's ability to predict SSI, the authors have not clinically used the absolute value of either test following completion of this study but rather rely on the trend of the patient's SAA values. As there is variation in the absolute value of SAA related to degree of initial trauma as well as surgical trauma, the trend has been more indicative of the presence of subclinical infection than the values at individual time points. Based on the results seen in this study, if SAA does not decline in the early postoperative period between days 3 and 5, it is likely that there is a subclinical SSI present. With the median time to development of clinical signs of SSI in cases undergoing internal fixation having been shown in a previous report from this institution to be 9.5 days, earlier detection of SSI using serial SAA monitoring is possible in some cases and SAA values may aid in determining the significance of early clinical signs of SSI (2). SAA changes more rapidly in response to patient status compared to fibrinogen, however, in the absence of the ability to measure SAA, fibrinogen may also be used as a marker for SSI. The cost of running SAA in our hospital is insignificant when compared with total costs for long bone fracture repair and arthrodesis.

Limitations of the study include the small number of cases and the criteria for determining positive SSI. Surgeons were blinded to the SAA and fibrinogen levels so determination of SSI should not have been skewed by an increase in acute phase proteins. Individual clinicians, as there were four evaluating cases during this study, may have evaluated some subjective measures for diagnosis of infection differently as there were not strict objective criteria to which they were required to adhere. The percentage of horses that developed surgical site infection was similar to previous reports from this institution (1, 2). However, not all qualifying cases admitted to the hospital during the time period of this study were included, and therefore this is not a complete case population. Cases were prospectively enrolled that were at a high risk for infection. Risk factors for surgical site infection were not within the scope of this study. It is possible that SAA may have been elevated for reasons unrelated to the surgical site or procedure. Other postoperative complications or infections not involving the surgical site were not recorded. Not all horses were included at each time point.

In conclusion, the current study demonstrated the value of postoperative monitoring of SAA, which could lead to earlier suspicion and therefore recognition of signs of SSI in horses. If SAA is persistently elevated on days 5–7 postoperatively, the possibility of a SSI could be investigated earlier and therefore treatment, such as continuation of administration of systemic antimicrobials and local delivery of antimicrobials by intravenous regional limb perfusions, can be instituted hopefully to achieve more successful outcomes.

## Data availability statement

The raw data supporting the conclusions of this article will be made available by the authors, without undue reservation.

## Ethics statement

The animal study was reviewed and approved by Institutional Animal Care and Use Committee of the University of Pennsylvania New Bolton Center. Written informed consent was obtained from the owners for the participation of their animals in this study.

## Author contributions

CT was responsible for data collection and manuscript preparation. DS was responsible for statistical analysis and manuscript revision. MM was responsible for data collection, study design, and manuscript revision. H-SC was responsible for data collection and manuscript revision. DR was responsible for study design and manuscript revision. DL was responsible for study design, data interpretation, and manuscript revision. All authors contributed to the article and approved the submitted version.

## Funding

Test kits were provided by Stablelab^®^.

## Conflict of interest

The authors declare that the research was conducted in the absence of any commercial or financial relationships that could be construed as a potential conflict of interest.

## Publisher's note

All claims expressed in this article are solely those of the authors and do not necessarily represent those of their affiliated organizations, or those of the publisher, the editors and the reviewers. Any product that may be evaluated in this article, or claim that may be made by its manufacturer, is not guaranteed or endorsed by the publisher.
